# Publishing in an open access age: preserving the scribbles, getting heard, and assuring the quality of information

**DOI:** 10.1002/brb3.5

**Published:** 2011-09

**Authors:** Andrei V Alexandrov


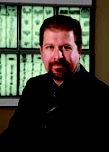


The act of publishing scientific research preserves our past achievements while laying a foundation for future progress. Consequently, assurance of quality is crucial to further scientific research, and it is imperative for leaders in the community to maintain appropriate standards while making publishing as dynamic as modern times require.

With the launch of this open access, multidisciplinary journal, we offer a broad scientific community a forum for rapid publication of original contributions, covering all aspects of neurology, neuroscience, psychology, and psychiatry. By offering quality assurance through stringent peer review and delivering content quickly, broadly, and effectively, we aim for *Brain and Behavior* to become both your regular source for neurology, neuroscience, psychology and psychiatry research, as well as a platform by which you will offer your best science to the broadest community possible.

At the launch of this Journal, my colleagues asked me: why yet another journal? And then: why should authors pay for their manuscripts to be published online?

It turns out that despite all of the journals available to scientists, publishing a strong manuscript can still be challenging. Many accomplished scientists can tell you stories of submitting their best papers to two or three different journals before they were finally accepted for publication.

Meanwhile, not all manuscripts feature groundbreaking, novel research; many papers contain solid experiments and clinical studies that merely serve to confirm others’ theories or experiments. These latter papers are important because even the best theories or break-through experiments must often wait for multiple confirmations before they are accepted by the scientific community. Unfortunately, space limitations and page budgets frequently force editors and reviewers to choose between novel, ground-breaking research and quality confirmatory studies, often at the expense of the latter.

This is where digital publishing comes into play. In the digital era, content becomes limited only by the device used to access it. Journals are now accessible on computers at institutional libraries, on laptops in homes, on mobile devices in coffee shops – virtually anywhere the internet is available. Access to the Web has granted scientists an unprecedented view of the world that will undoubtedly expand as future information technologies continue to tear down barriers.

The open access publishing model gives authors an opportunity to disseminate their results to an extremely wide audience in new ways. Authors can publicize their work knowing that readers can instantly access it without the need for institutional or personal subscriptions. Given this landscape and the fact that publishing in this digital age is evolving rapidly, it is anyone's guess as to where we will end up in the future. Nevertheless, one thing remains certain: scientists will still need a way to filter sources and ensure that information is trustworthy. Proliferation of various electronically published periodicals can mislead an ever-growing readership, and it is imperative for leaders in the scientific community to maintain the standards of peer-reviewed publications while making publishing as dynamic and advanced as possible.

I would like to congratulate Wiley for taking a proactive approach by putting professional resources behind their neurology, neuroscience, psychology, and psychiatry family of journals and moving forward with open access publishing. We debated at length (and still do with our Editorial Board and the Publisher) how best to proceed, and your feedback as authors and readers is very important. Please let us know your thoughts and suggestions through the Journal website.

Together with my Associate Editors, our multidisciplinary Editorial Board, and Editors of participating Wiley-published journals, I look forward to developing this open access journal, *Brain and Behavior*.

